# Dexmedetomidine for prevention of postoperative delirium in elderly patients through hypoxia-inducible factor: A narrative review

**DOI:** 10.1097/MD.0000000000046368

**Published:** 2025-12-19

**Authors:** Jie Zhang, Mengjun Wu, Li Li, Yichao Wang

**Affiliations:** a Outpatient Department, West China Hospital, Sichuan University, Chengdu, Sichuan Province, China; b Division of Thyroid Surgery, Department of General Surgery, West China Hospital, Sichuan University, Chengdu, Sichuan Province, China; c West China School of Nursing, Sichuan University, Chengdu, Sichuan Province, China; d Department of Anesthesiology, Chengdu Women’s and Children’s Central Hospital, University of Electronic Science and Technology, Chengdu, Sichuan Province, China; e Laboratory of Thyroid and Parathyroid Disease, Frontiers Science Center for Disease-Related Molecular Network, West China Hospital, Sichuan University, Chengdu, Sichuan Province, China.

**Keywords:** aged, anesthesia, delirium, dexmedetomidine, hypoxia-inducible factor 1 (HIF-1), neuropsychological tests, postoperative delirium, surgery

## Abstract

Postoperative delirium (POD) remains a significant clinical concern, particularly in elderly surgical patients, where it is associated with increased morbidity, prolonged hospitalization, and elevated mortality. While the exact mechanisms underlying POD are not fully understood, cerebral desaturation during general anesthesia maintenance has been identified as an independent risk factor, especially in aging populations. Hypoxia triggers the expression of hypoxia-inducible factor-1 (HIF-1), which plays a dual role in cerebral hypoxia: it exhibits neuroprotective effects by regulating neuronal apoptosis pathways in neonatal hypoxia-ischemia models and promotes brain repair through hypoxia-responsive mechanisms. However, anesthetics have been shown to suppress HIF-1 activity in elderly patients, potentially exacerbating neuronal vulnerability. Dexmedetomidine (Dex) demonstrates neuroprotective properties by modulating the HIF-1α pathway, as evidenced by its ability to reduce neuronal apoptosis and ameliorate cerebral ischemia-reperfusion injury in animal models. These findings suggest that Dex mitigates postoperative delirium by counteracting anesthetic-induced HIF-1α suppression and maintaining neuronal integrity. Consequently, targeting the HIF-1α pathway emerges as a promising therapeutic strategy for preventing POD in elderly patients, offering potential avenues for both prophylaxis and treatment. This review elucidates the involvement of HIF-1 in POD pathogenesis and explores innovative approaches to mitigate delirium through Dex-mediated HIF-1α modulation in aging populations.

## 1. Introduction

Postoperative delirium (POD) is a serious complication in elderly surgical patients, associated with prolonged hospitalization, morbidity, and mortality.^[1–5]^Clinical studies have consistently demonstrated that postoperative delirium is associated with significant adverse outcomes, including increased morbidity, elevated 30-day mortality rates, and prolonged hospital stays by an average of 3 days.^[2]^ The pathophysiology of delirium is multifactorial, with several potential mechanisms contributing to its development, such as systemic inflammatory response syndrome, neuroinflammation,^[3–5]^ and disturbances in brain metabolism.^[6–8]^ Current evidence-based strategies for perioperative delirium prevention include the implementation of anesthesia depth monitoring, intraoperative administration of dexmedetomidine, and multimodal analgesic approaches.^[6,7]^ Despite these advancements, the precise mechanisms underlying postoperative delirium, particularly in elderly patients, remain poorly understood and require further investigation.

Cerebral desaturation (hypoxia) is a clinically significant independent risk factor for postoperative delirium, with regional cerebral oxygen saturation (rSO2) monitoring studies demonstrating its occurrence in more than 20% of noncardiac surgery cases.^[4–6]^ This hypoxic condition activates hypoxia-inducible factor-1 (HIF-1), a master transcriptional regulator orchestrating cellular responses to oxygen deprivation.^[8–12]^ As a central mediator of hypoxic adaptation, HIF-1 exerts critical regulatory functions across diverse pathological processes, including neonatal hypoxic-ischemic brain injury.^[9]^ Experimental evidence reveals that neuron-specific HIF-1 deficiency exacerbates ischemic brain damage, whereas augmented HIF-1α expression correlates with enhanced neural functional recovery in rodent stroke models.^[10,11]^ Recent investigations highlight that perioperative anesthetic agents and medications can profoundly modulate HIF-1 activity.^[13–15]^ Notably, barbiturates including thiopental and thiamylal, demonstrate dose-dependent reversible suppression of hypoxia-induced HIF-1 activation in both neuronal and non-neuronal cell populations, effectively attenuating HIF-1-mediated transcriptional responses and downstream gene expression.^[11–14]^ Furthermore, hippocampal HIF-1α/vascular endothelial growth factor signaling has emerged as a key molecular mechanism underlying isoflurane-associated cognitive impairment, suggesting a novel therapeutic avenue for preventing and managing postoperative delirium.^[15]^ Collectively, these mechanistic insights support our central hypothesis that HIF signaling constitutes a pivotal pathogenic pathway in postoperative emergence delirium, particularly among elderly surgical populations.

Dexmedetomidine (Dex), a potent and highly selective α2-adrenoreceptor agonist, is widely recognized for its anxiolytic and sedative properties. Clinical studies have demonstrated that prophylactic administration of Dex significantly reduces the incidence of postoperative delirium following surgical procedures.^[16]^ Extensive animal and experimental research has further elucidated the neuroprotective mechanisms of Dex, suggesting that it may attenuate neuronal apoptosis induced by ischemia-reperfusion (I/R) injury through inhibition of the HIF-1α pathway, thereby ameliorating cerebral I/R injury.^[16,17]^

Based on these findings, we hypothesize that HIF-1α plays a pivotal role in the pathophysiology of postoperative delirium in elderly patients and that Dex may exert its preventive effects through modulation of the HIF-1α signaling pathway. This review explores the hypothesis that dexmedetomidine may prevent POD in elderly patients by modulating HIF-1α pathways to provide novel insights into potential therapeutic targets for the prevention and treatment of Dex-induced postoperative delirium in geriatric patients.

This narrative review adhered to SANRA (Scale for the Assessment of Narrative Review Articles) guidelines. Literature searches were conducted in PubMed, EMBASE, and Cochrane Library (January 1990–December 2023) using keywords: dexmedetomidine, postoperative delirium, HIF-1α, elderly, and anesthesia. Inclusion criteria encompassed human/animal studies in English. Two authors (J.Z. and W.M.J.) independently screened titles/abstracts; full-text eligibility was assessed by Y.W. and L.L. Discrepancies were resolved by consensus. The report was approved by the Institutional Review Board of West China Hospital, Sichuan University.

## 2. Anesthesia-induced cerebral hypoxia serves as a critical mediator in the pathogenesis of postoperative delirium

Cerebral hypoxia represents a significant risk factor for perioperative delirium,^[18]^ with anesthetic agents being a potential contributor to its occurrence. The incidence and severity of cerebral hypoxia vary depending on the type of anesthetic administered. Clinical studies have consistently demonstrated the occurrence of cerebral desaturation during general anesthesia,^[19]^ with procedural sedation being associated with hypoxemic events in up to 21% of patients.^[20]^ The impact of anesthetics on cerebral oxygen metabolism varies significantly between agents, as evidenced by the higher frequency of cerebral desaturation during cardiopulmonary bypass in patients receiving fentanyl compared to those administered propofol.^[21]^ The mechanism underlying anesthetic-induced cerebral hypoxia is primarily attributed to their cardiovascular depressant effects. This phenomenon is particularly relevant in elderly surgical patients, where cerebral hypoxia may contribute to the pathophysiology of postoperative cognitive decline.^[21]^

Emerging evidence demonstrates that anesthetic-induced cerebral hypoxia plays a pivotal role in the development of postoperative neurocognitive disorders across diverse patient populations.^[21–23]^ Clinically significant cerebral oxygen desaturation during surgery correlates with both immediate postoperative cognitive decline^[22–24]^ and persistent neuropsychological deficits, particularly when volatile anesthetics are combined with sedative agents.^[25]^ This phenomenon exhibits age-specific patterns, with pediatric patients showing heightened susceptibility to anesthesia-induced cerebral metabolic suppression,^[26]^ while neonates undergoing thoracoscopic procedures experience particularly profound pathological desaturation events during visceral manipulation. The cumulative effect of these oxygen metabolic disturbances may contribute to the well-documented association between anesthesia exposure and long-term neurological sequelae.^[15]^

## 3. Roles of HIF in cerebral hypoxia

Hypoxia triggers the activation of numerous genes, with hypoxia-inducible factor-1 (HIF-1) serving as a central regulatory protein in this process. HIF-1 is a heterodimeric transcription factor composed of two subunits: the oxygen-sensitive HIF-1α and the constitutively expressed HIF-1β.^[24–27]^ Under hypoxic conditions, the inactivation of prolyl hydroxylase domain (PHD) enzymes leads to the stabilization and subsequent nuclear translocation of HIF-1α, where it dimerizes with HIF-1β to form the active transcription complex.^[28]^ This complex regulates the expression of genes critical for maintaining cellular oxygen homeostasis, including those involved in oxygen utilization, erythropoiesis, angiogenesis, and mitochondrial function.^[29]^ These adaptive responses may confer resistance to hypoxia- or ischemia-induced neurological injury.

The HIF signaling pathway plays a crucial role in endogenous neuroprotection.^[30]^ Experimental evidence demonstrates that pharmacological upregulation of HIF-1α through desferoxamine pretreatment confers protection against cerebral ischemia,^[31]^ while neuron-specific HIF-1 deficiency exacerbates ischemic brain injury.^[32]^ Furthermore, targeted downregulation of neuronal HIF-1α has been shown to increase susceptibility to cerebral ischemia-induced brain damage.^[31–33]^ Under moderate hypoxic conditions, HIF-1 stabilization promotes the expression of protective genes; however, prolonged hypoxia leads to pathological HIF-1α stabilization, which is associated with increased p53 activity and the activation of deleterious gene programs.^[34,35]^ Given these mechanisms, we propose that HIF-1 likely plays a crucial protective role during surgical procedures in elderly patients, potentially serving as a key mediator of neuroprotection in this vulnerable population.

## 4. Anesthetics alter HIF-1α signaling in elderly patients through dose-dependent regulation of nuclear translocation and protein stability

Intraoperative cerebral oxygen desaturation, a frequent occurrence during general anesthesia, has been consistently associated with the development of postoperative cognitive dysfunction.^[34–38]^ The cerebral cellular response to hypoxic conditions is primarily mediated by hypoxia-inducible factors (HIFs), a family of transcription factors that regulate the expression of numerous genes involved in cellular adaptation to low-oxygen environments. While HIF-hydroxylase activity and HIF-1 stability remain unaffected by anesthetic agents, studies have demonstrated that general anesthetics can significantly inhibit HIF-1 protein synthesis.^[39]^ Specifically, the intravenous anesthetic propofol has been shown to suppress HIF-1α activation induced by either hypoxia or cobalt chloride (CoCl_2_) stimulation. In the context of cerebral I/R injury, the volatile anesthetic sevoflurane exhibits multiple protective effects, including reduction of inflammatory factors, inhibition of apoptosis, and downregulation of both HIF-1 and heat shock protein 70 (HSP70) expression.^[40]^ Furthermore, “Propofol and barbiturates inhibit HIF-1α transcriptional activity in neuronal cells (IC₅₀: 50–100 μM).^[41,42]^ However, evidence in elderly models remains limited. Aged rodents show 40% greater HIF-1α suppression by propofol than young counterparts,^[43]^ possibly due to blood-brain barrier alterations. Clinically, correlations between anesthetic dose, cerebral hypoxia duration, and POD risk require further investigation.”

## 5. Dexmedetomidine inhibit the neuronal apoptosis by inhibiting the HIF‐1α pathway

Dexmedetomidine (Dex), a potent and highly selective α2-adrenergic receptor agonist, exhibits a wide range of pharmacological properties, including sedative, analgesic, anti-inflammatory, antioxidant, and antiapoptotic effects.^[43]^ Substantial evidence has demonstrated the neuroprotective and antiapoptotic properties of Dex in various I/R injury models. Specifically, preclinical studies have shown that Dex plays a crucial protective role in both cerebral and cardiac I/R injury, effectively mitigating focal ischemia in rabbit models and reducing cardiac ischemia-reperfusion injury in rat models.^[16,42,43]^ These findings underscore the therapeutic potential of Dex in managing I/R-related tissue damage across different organ systems.

Animal studies demonstrate dexmedetomidine inhibits neuronal apoptosis via HIF-1α suppression (*P* < .01).^[43,44]^ Emerging research demonstrates that HIF-1α plays a pivotal role in modulating apoptotic pathways across diverse brain injury models, particularly in cerebral ischemia and traumatic brain injury. The hypoxia-inducible factor exhibits context-dependent regulatory effects on cell survival and death, with its activity being intricately linked to oxygen availability and metabolic stress responses.^[43–45]^

Notably, pharmacological blockade of the HIF-1α signaling pathway using 2-methoxyestradiol (2ME2) has been demonstrated to enhance the neuroprotective effects of dexmedetomidine (Dex) through augmented apoptosis inhibition.^[46,47]^ In I/R rat models, the activation of HIF-1α appears to counteract the antiapoptotic effects of Dex, suggesting an antagonistic relationship between HIF-1α signaling and Dex-mediated neuroprotection.^[48,49]^ The neuroprotective effects of Dex against cerebral I/R injury are mediated through its dual action of inhibiting neuronal apoptosis and suppressing HIF-1α expression. These findings suggest that Dex exerts its protective effects against cerebral I/R injury primarily through the inhibition of both the HIF-1α pathway and downstream apoptotic mechanisms in the I/R rat brain.

## 6. Conclusion

The study reported that prophylactic dexmedetomidine significantly decreased the occurrence of postoperative delirium after surgery. Postoperative delirium, characterized by psychomotor agitation and cognitive disturbances, represents a particularly challenging complication in elderly patients. Based on the current findings, we hypothesize that general anesthetics may downregulate both the expression and functional activation of hypoxia-inducible factor (HIF) proteins in the brain, potentially intensifying HIF-1-mediated responses to cerebral hypoxia, particularly in aged patients. We propose a plausible yet unvalidated mechanism: Dexmedetomidine may mitigate POD in elderly patients by normalizing maladaptive HIF-1α responses to perioperative cerebral hypoxia. However, key limitations include reliance on preclinical data, heterogeneity in delirium assessment tools across cited studies, and undetermined dose-response relationships. Future research should: Validate HIF-1α as a POD biomarker in human CSF/serum and Conduct RCTs correlating dexmedetomidine infusion rates with HIF-1α activity and neurocognitive outcomes.

## Author contributions

**Data curation:** Li Li.

**Methodology:** Mengjun Wu.

**Resources:** Li Li.

**Supervision:** Yichao Wang.

**Validation:** Yichao Wang.

**Visualization:** Mengjun Wu, Yichao Wang.

**Writing – original draft:** Jie Zhang.

**Writing – review & editing:** Li Li.
